# Changing pattern of the detection of locoregional relapse in breast cancer: the Edinburgh experience

**DOI:** 10.1038/sj.bjc.6603815

**Published:** 2007-05-29

**Authors:** D A Montgomery, K Krupa, W J L Jack, G R Kerr, I H Kunkler, J Thomas, J M Dixon

**Affiliations:** 1 Clinical Research Fellow, University Department of Surgery, Level 2, Queen Elizabeth Building, Glasgow Royal Infirmary, Glasgow, G31 2ER, Scotland, UK; 2 Department of Surgery, Paisley Royal Alexandra Hospital, Paisley, PA2 9PN, Scotland, UK; 3 Academic Office, Edinburgh Breast Unit, Western General Hospital, Edinburgh, EH4 2XU, Scotland, UK; 4 Department of Clinical Oncology, Edinburgh Cancer Centre, Western General Hospital, Edinburgh, EH4 2XU, Scotland, UK; 5 Department of Pathology, Western General Hospital, Edinburgh, Scotland, UK

**Keywords:** breast cancer, recurrence, detection, retrospective review, survival

## Abstract

The guidelines for follow-up of breast cancer patients concentrate on the first 3–5 years, with either reduced frequency of visits or discharge after this. They also recommend mammography, but no evidence exists to inform frequency. We analyse treatable relapses in our unit from 1312 patients with early stage breast cancer treated by breast conserving surgery (BCS) and postoperative radiotherapy between 1991 and 1998 to assess appropriateness of the guidelines. A total of 110 treatable relapses were analysed. Treatable relapse developed at 1–1.5% per year throughout follow-up. Forty-eight relapses were in ipsilateral breast, 25 ipsilateral axilla, 35 contralateral breast, 2 both breasts simultaneously. Thirty-seven relapses (33.5%) were symptomatic, 56 (51%) mammographically detected, 15 (13.5%) clinically detected, 2 (2%) diagnosed incidentally. Mammography detected 5.37 relapses per 1000 mammograms. Patients with symptomatic or mammographically detected ipsilateral breast relapse had significantly longer survival from original diagnosis (*P*=0.0002) and from recurrence (*P*=0.0014) compared with clinically detected. Treatable relapse occurs at a constant rate for at least 10 years. Clinical examination detects a minority (13.5%). Relapse diagnosed clinically is associated with poorer outcome. Long-term follow-up based on regular mammography is warranted for all patients treated by BCS.

Historically, follow-up in breast cancer has focused on the early detection of relapse. The approach to detecting locoregional and metastatic relapse has changed as understanding of the natural history of the disease has improved. The diagnosis of incurable metastatic disease when patients are still asymptomatic does not significantly alter outcome (Del Turco *et al*, 1994; The GIVIO investigators, 1994). Moreover, regular visits are likely to increase distress and reduce quality of life, and may lead to unnecessary treatment (Del Turco *et al*, 1994; The GIVIO investigators, 1994). Tests to detect metastatic disease are no longer recommended (National Institute for Clinical Excellence, 2002).

Unlike metastatic relapse, it is generally accepted that, particularly in patients who have had breast-conserving surgery, locoregional relapse is potentially treatable and should be detected early (Clark *et al*, 1985; Recht *et al*, 1989; Fowble *et al*, 1990; Kurtz *et al*, 1990; Haffty *et al*, 1991; Dalberg *et al*, 1998). Regular periodic mammography and clinical examination are performed to detect asymptomatic ipsilateral and contralateral disease. However, in a recent meta-analysis routine clinic visits were at best inefficient in the detection of treatable locoregional relapse (de Bock *et al*, 2004). We have previously reported that only one-third of these are detected by clinicians at routine visits in asymptomatic patients, representing one relapse detected for every 175 routine clinic visits (Jack *et al*, 1998). Despite locoregional relapse being relatively rare, and most such relapses being detected by other means, many clinicians still consider routine clinical examination necessary for detection of relapse. Increasingly, less frequent or shorter follow-up is being advised in an attempt to improve efficiency. While reduced follow-up has been shown to be acceptable to patients (Gulliford *et al*, 1997), little is known of its impact on loco-regional relapse detection and overall survival.

We have examined the pattern of treatable relapse in our unit, particularly with regard to its timing and method of detection, relating our findings to current guidelines to highlight their likely effect on our patients.

## PATIENTS AND METHODS

Between 1991 and 1998, 1312 patients were treated for early stage breast cancer by breast conservation surgery, axillary node sampling or clearance and postoperative radiotherapy to the breast +/− ipsilateral lymphatics. Systemic therapy was given according to local and national guidelines. Follow-up was shared between the Edinburgh Breast Unit and the Department of Clinical Oncology, Western General Hospital, Edinburgh.

Although initial clinical follow-up consisted of 3–4 monthly visits for the first 2 years, 6 monthly for 3 years then annual visits until the tenth anniversary, from 2000, all patients were changed to annual follow-up visits only. Throughout, all patients were instructed in regular breast self-examination. Additional interval visits for assessment were arranged by patients, their general practitioner or other health-care professionals as required. Patients were usually discharged to the national breast screening unit at 10 years. Annual bilateral mammography was undertaken throughout. Median follow-up was 10 years, range 1.5–15 years.

Characteristics of the included patients are presented in Table 1.

Locoregional relapse was defined as any relapse in the ipsilateral breast and axilla or the contralateral breast. Contralateral breast cancers are included here as the risk of subsequent contralateral breast cancer in patients who have had a previous breast cancer is 3–5 times greater than the population risk (Mellink *et al*, 1991). As such, detecting new contralateral cancers is an important component of breast cancer follow-up programmes. Supraclavicular relapse was included with metastatic disease. Detection of metastatic disease within 3 months of locoregional relapse was considered to constitute simultaneous local and distant relapse and these patients were excluded from the analysis.

A list of all patients known to have suffered an isolated locoregional relapse or new contralateral cancer was generated from our database in January 2006. A retrospective study of these case records was then undertaken. Time from diagnosis to relapse, method of detection of relapse (routine mammography, routine clinical examination in an asymptomatic patient or investigation of a patient who attended complaining of relevant symptoms) and type of clinic (routine or interval) was recorded. Time from relapse to development of metastatic disease or death was then calculated and survival was compared for each mode of detection. For all relapses detected clinically in the first instance, we established whether the relapse was also visible on mammography.

## RESULTS

**Figure 1:**
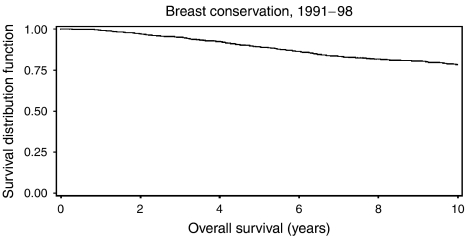
Overall survival.

**Figure 2:**
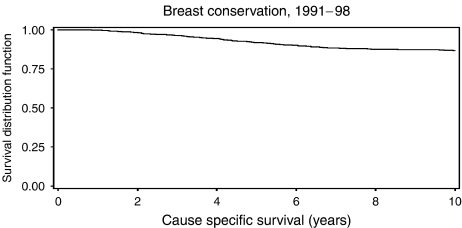
Cause specific survival.

**Figure 3:**
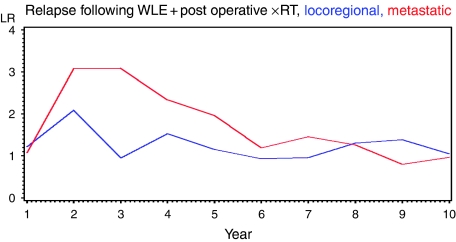
Annual incidence of relapse per 100 women at risk.

Between 1991 and 1998, 1312 patients were treated for primary operable breast cancer by breast conservation surgery. Figure 1 displays overall survival, Figure 2 cause specific survival. The overall 5-year survival was 89.2%; 10-year survival was 77.2%. Not included in the analysis were 31 patients with synchronous locoregional and metastatic disease and 11 with metastatic disease before locoregional relapse; these are included as metastatic relapse in Figure 3 (see below). A total of 116 patients suffered an isolated locoregional relapse. After exclusion of four lacking available records, and two lost to follow-up through moving away from the area, 110 patients were left for analysis. Details of all patients with treatable relapse are shown in Table 2.

48 patients developed ipsilateral breast relapse, 11 with concomitant axillary disease. Twenty-five patients had isolated ipsilateral axillary relapse and 35 developed new contralateral cancers. One patient developed ipsilateral breast and axillary relapse and a new contralateral cancer simultaneously and one developed ipsilateral breast relapse and a new contralateral cancer simultaneously.

The recurrence rate in this cohort is presented as Figure 3. The incidence of metastatic relapse peaks at just over 3% per annum at 2–3 years and remains above 2% per year for up to 5 years before falling off. In contrast, the incidence of locoregional relapse remains constant at 1–1.5% over the whole follow-up period.

Site of relapse and method of detection are summarised for the 110 patients in Table 3, together with the numbers who died (in brackets).

### Interval appointments

Twenty-three of the 110 patients (21%) had relapse detected at an interval clinic visit of whom 22 complained of relevant symptoms, and the other had an abnormality noticed by careers. Of these 23 patients, 10 had ipsilateral breast relapse, 6 axillary relapse and 2 had synchronous breast and axillary relapse, with symptoms from both sites. Five patients discovered their new contralateral cancer.

Two patients (2%) were referred back to clinic having been discharged from further follow-up at 10 years post-treatment. One complained of symptoms related to axillary relapse (included in Table 3 as having been diagnosed at an interval clinic with symptoms) and one had abnormal routine breast screening mammograms, which indicated breast and axillary relapse (included in Table 3 as routine mammographically detected relapse).

### Routine appointments

The remaining 85 relapses (77%) were detected at routine clinics or by routine mammography. The pattern of relapse and method of detection for this group is also presented in Table 3.

Of the four ipsilateral breast recurrences diagnosed clinically, the most recent annual surveillance mammogram was negative in two. Mammography was repeated and was negative in the third. In the fourth, mammography did reveal some distortion, but this was reported as benign post-surgical changes in their first routine follow-up mammogram 1 year after surgery. Neither of the two contralateral cancers detected clinically were visible on mammography. Of the nine axillary relapses diagnosed clinically, one also visible on mammography was included as a clinical diagnosis as it was diagnosed at a routine appointment at which mammography was not scheduled.

In total, 37 relapses (33.5%) were symptomatic, 56 (51%) mammographically detected, 15 (13.5%) clinically detected and 2 (2%) were diagnosed incidentally.

A total of 10 415 mammograms were undertaken during follow-up, with 56 treatable relapses diagnosed that is 5.37 treatable relapses per 1000 mammograms.

### Survival

Details of the 37 deaths among the 110 patients during the follow-up period are shown in Table 3.

#### Ipsilateral breast

**Figure 4:**
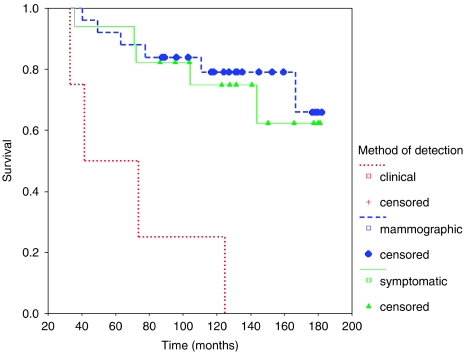
Survival from original operation in patients with ipsilateral breast relapse.

Forty-eight patients suffered ipsilateral breast recurrence, of whom 11 had additional axillary recurrence. Overall 5-year survival was 87.5% from original operation, and 64% from diagnosis of recurrence. Survival by method of detection is shown below (Figure 4).

**Figure 5:**
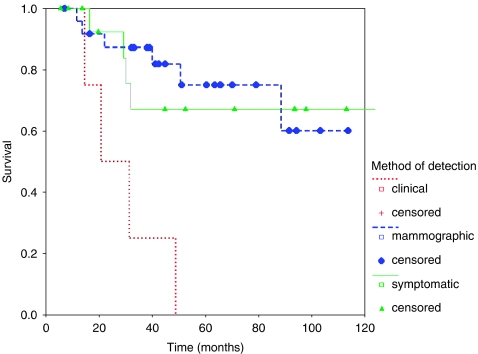
Survival from time of relapse in patients with ipsilateral breast relapse.

Overall survival was significantly reduced among those with recurrence diagnosed clinically compared with either other method (log rank 2 df *P*=0.0002). This remains highly significant even if cases with axillary disease are excluded (*P*=0.0004), and reflects a significantly longer survival from recurrence (Figure 5) (log rank 2 df *P*=0.0014) rather than a difference in time to recurrence detection.

The pathological characteristics of the recurrent disease are presented as Table 4. Mean age at recurrence is also shown. Nodal status was available for all 48 patients. Size of the recurrent lesion was unavailable for 10 women, with grade of the recurrence unavailable for 13. Receptor status was seldom available. Trends for those with clinically detected relapse to be older than others, and for mammographically detected lesions to be smallest at detection, with symptomatic largest, were not significant. Overall, there was no significant difference in any of the clinicopathological features in relation to how relapse was detected, and Nottingham Prognostic Index of the relapse was similar for all three groups.

#### Contralateral breast

There was no association between method of detection of relapse and survival in patients who developed a new contralateral breast cancer. However, 25 out of 35 of these (71%) were diagnosed by mammography with 8 self-diagnosed and 2 picked up on clinical examination. Both of the patients with new cancers detected clinically were well at last follow-up. Overall 5-year survival from time of relapse for all patients with contralateral breast relapse was 81%.

#### Ipsilateral axilla

There was similarly no association between method of detection of recurrence and survival in patients who had isolated ipsilateral axillary relapse, although the numbers overall were small. Overall 5-year survival from time of relapse for patients with axillary relapse was 61%.

## DISCUSSION

The most recent guidelines for the follow-up of breast cancer patients from the American Society of Clinical Oncology (ASCO) recommend 3–6 monthly follow-up for 3 years, 6–12 monthly for 2 years then annual follow-up (Khatcheressian *et al*, 2006). No advice is given regarding discharge, although 1999 guidelines recommended this at 10 years (American Society of Clinical Oncology, 1999). In Canada, the Steering Committee guidelines state that there is no compelling evidence to support any particular frequency of clinic visits, but concludes that visits should be more frequent initially due to a higher risk of recurrence in the first few years, reducing to annual after 3–5 years and being continued indefinitely (The Steering Committee on Clinical Practice Guidelines for the Care and Treatment of Breast Cancer, 1998). Annual mammography is recommended by both groups, although ASCO acknowledge the lack of good evidence (Khatcheressian *et al*, 2006).

In contrast, the recent guidelines for England and Wales (NICE) recommend follow-up of only 2–3 years in total, but leave frequency of visits open (National Institute for Clinical Excellence, 2002). The British Association of Surgical Oncology (BASO) recommends slightly longer follow-up, with discharge at 5 years, again leaving frequency of visits open (The Association of Breast Surgery @ BASO and Royal College of Surgeons of England, 2005). Neither group recommends how often mammography should be performed, with NICE stating that evidence-based protocols should be agreed locally, and BASO referring to the Royal College of Radiologists advice that mammography should be every 1–2 years (The Royal College of Radiologists, 1995).

Although these guidelines appear to vary markedly, all assume that relapse is commonest in the first few years after treatment. A greater hazard rate for relapse during the first 3 years was just evident in the ATAC trial (The ATAC Trialists' Group, 2005), but reflects a higher rate of distant relapse in this period. Others have reported that the rate of locoregional relapse is initially high, and then falls off, so that most treatable relapses occur in the first 3–5 years (Elder *et al*, 2006). However, these studies do not include new contralateral cancers. Other studies have a very brief median follow-up, without analysis of recurrence over the long term (Donnelly *et al*, 2001). The present study confirms that rate of distant relapse peaks in the first 5 years, but this is not mirrored by the pattern of locoregional relapse, which remains constant at 1–1.5% for at least 10 years. Others have also reported that while the incidence of true local recurrence declines with time, that of new breast cancers in other areas of the previously treated breast and new contralateral breast cancers increases with time, so that the overall rate of treatable relapse remains constant for over 15 years (Freedman *et al*, 2005).

The second assumption in various guidelines is that clinical examinations are of particular value, with more frequent visits recommended during the perceived high-risk period of the first few years. Previous studies have consistently shown that multiple clinical visits do little to increase yield and only serve to reduce the ‘cost effectiveness’ of follow-up (de Bock *et al*, 2004). Our data confirm this. In 10 years of follow-up in 1312 women, only 15 relapses were detected clinically. This is a very low yield. Mammography in contrast makes a much larger and more significant contribution to relapse detection, not only in the area of contralateral new breast cancer detection but also in detecting ipsilateral breast recurrence. In fact, a detection rate of 5.37 cancers per 1000 mammograms is higher than that reported as recently as 2003 by the breast screening service in the UK that carries out 3-yearly mammography (NHS Cancer Screening Programmes, 2003), and is equivalent to that in this body's latest (2007) report.

Why patients with clinically detected relapse do less well is unclear from this study, and although slightly older on average, the difference was not significant. Nottingham Prognostic Index for the relapse was similar for all three methods of detection.

Our data reveal that the basic assumptions behind the guidelines for follow-up are incorrect. If a central aim is the detection of treatable relapse, as stated in the guidelines, there is no justification for focussing on the first 2–3 years after treatment; treatable relapse occurs at a constant rate over at least 10 years. Our data suggest that clinical examinations do not improve outcome. Women are well schooled in self-surveillance of their breasts. Axillary relapse, on the contrary, is more frequently detected by clinicians, 36% being so detected. However, isolated axillary relapse was relatively rare, with just 25 in 1312 patients over 10 years, and only nine clinically detected relapses. We intend to highlight the need for regular axillary self-examination, not previously stressed, and hope this will further improve the yield of self-examination, particularly in this era of sentinel lymph node biopsy.

Detection of treatable relapse is only one of the aims of follow-up. Additional aims concern psychological problems and adverse effects of therapy, particularly lymphoedema (National Institute for Clinical Excellence, 2002). It is uncertain whether routine clinic visits achieve these aims. While women do find clinics reassuring when told they do not have recurrence (Morris *et al*, 1992; Kiebert *et al*, 1993), visits are also attended by great anxiety (Paradiso *et al*, 1995). There is little doubt that clinicians are not good at detecting psychological problems in the clinical setting (Hardman *et al*, 1989; Passik *et al*, 1998) and patients are reluctant to report such psychological problems themselves (Valente *et al*, 1994; Pennery and Mallet, 2000). Certainly, one study has shown no reduction in quality of life among women randomised to receive no routine clinic visits compared with regular visits (Gulliford *et al*, 1997).

Whether routine clinics are necessary for the detection and treatment of adverse effects of therapy is also uncertain. Detection of lymphoedema is given central importance in the NICE guidelines, although its symptoms do not correlate with objective measurements of arm swelling (Schunemann and Willich, 1998). Moreover, there is little in the way of an effective therapy. Side effects of treatment might be detected better by education and focussing follow-up on the symptomatic individual.

Aromatase inhibitors have introduced a new consideration in follow-up. The NICE guidelines only mention adjuvant hormonal therapy to say that the general practitioner should be responsible for stopping it at 5 years (National Institute for Clinical Excellence, 2002). With recent indications for switch and extended aromatase treatments, which are instituted in specialist care, discharge to general practice after 3 years would not be appropriate. Moreover, hormonal therapy can significantly impact patients' overall well being, particularly in the first 3 months of therapy (Fallowfield *et al*, 2004). Patients should have access to specialist advice to help ameliorate any symptoms. Long-term rapid and flexible access to specialist care would be ideal in the face of new developments in breast cancer treatment, and emphasis should be placed on psychological and physical well-being. It is also likely that new hormonal therapies will alter the pattern of relapse so that even fewer relapses occur in the first 3 years after treatment (The ATAC Trialists' Group, 2005).

## Figures and Tables

**Table 1:** Study population

**Age (years)**	
Mean	56 years
Range	24–91 years
	
Tumour stage	No. of patients
T1	725
T2	587
	
Node status	No. of patients
Positive	354
Negative	958

**Table 2:** Study population, original pathology

**Age (years)**	
Mean	54.28 (s.d. 12.32)
Range	24–83
	
Tumour stage	No. of patients
T1	67 (inc. 1 bilateral)
T2	46
	
Node status	No. of patients
Positive	29
Negative	84 (inc. 1 bilateral)
	

**Table 3:** Pattern of relapse and detection

**Site of relapse**	**Symptoms**	**Clinical finding**	**Mammogram**	**Interval clinic with symptoms**	**Total**
Ipsilateral Breast	5 (1)	3 (3)	18 (5)	10 (3)	36 (12)
Ipsilateral Axilla	5 (5)	9 (3)	4 (1)	7 (2)	25 (11)
Ipsilateral breast and axilla	0 (0)	1 (1)	7 (1)	2 (1)	10 (3)
Contralateral breast	3 (2)	2 (0)	25 (5)	5 (1)	35 (8)
Ipsilateral axilla and bilateral breast	0	0	1 (0)	0	1 (0)
Bilateral breast	0	0	1 (1)	0	1 (1)
Total	13 (8)	15 (7)	56 (13)	24 (7)	108 (35)

The number of patients who subsequently died is included in brackets.

Two patients, not included in this table, had ipsilateral breast relapse, one node positive, diagnosed incidentally during breast reshaping procedures. Both of these patients subsequently died.

**Table 4:** Clinicopathological features of recurrence by method of detection

**Symptoms**	**Clinical finding**	**Mammography**
*Age of patient at relapse*		
57.7 (s.d. 14.7 years)	62.77 (s.d. 17.7)	59.74 (s.d. 11.8)
		
Mean size of relapse (mm)		
25.85 (s.d. 26.38)	17.67 (s.d. 10.97)	12.71 (s.d. 8.53)
		
*Grade of relapse*		
Grade 3: 9	Grade 3: 5	Grade 3: 2
Grade 2: 2	Grade 2: 10	Grade 2: 0
Grade 1: 2	Grade 1: 3	Grade 1: 1
Missing: 4	Missing: 7	Missing: 1
		
*Proportion of relapses with positive lymph nodes*		
2 of 17 (8.5%)	1 of 4 (25%)	7 of 25 (28%)
		
*Mean NPI*^a^ *of recurrence*		
4.36 (s.d. 1.33)	4.02 (s.d. 1.26)	3.87 (s.d. 1.16)

a NPI=Nottingham Prognostic Index.

## References

[bib1] American Society of Clinical Oncology (1999) Update of recommended breast cancer surveillance guidelines. J Clin Oncol 17: 1080–1082 10071303 10.1200/JCO.1999.17.3.1080

[bib2] Clark DH, Le MG, Sarrazin D, Lacombe MJ, Fontaine F, Travagli JP, May-Levin F, Contesso G, Arriagada R (1985) Analysis of loco-regional relapses in patients with early breast cancers treated by excision and radiotherapy: experience of the institut Gustave-Roussy. Int J Radiat Oncol Biol Phys 11: 137–145 2981790 10.1016/0360-3016(85)90372-4

[bib3] Dalberg K, Mattsson A, Sandelin K, Rutqvist LE (1998) Outcome of treatment for ipsilateral breast tumour recurrence in early-stage breast cancer. Breast Cancer Res Treat 49: 69–78 9694613 10.1023/a:1005934513072

[bib4] de Bock GH, Bonnema J, van Der Hage J, Kievit J, van de Velde CJH (2004) Effectiveness of routine visits and routine tests in detecting isolated locoregional recurrences after treatment for early stage invasive breast cancer: a meta analysis and systematic review. J Clin Oncol 22: 4010–4018 15459225 10.1200/JCO.2004.06.080

[bib5] Del Turco MR, Palli D, Carridi A, Ciatto S, Pacini P, Distante V (1994) Intensive diagnostic follow-up after treatment of primary breast cancer. A randomized trial. National Research Council Project on Breast Cancer Follow-up. JAMA 271: 1593–1597 7848404 10.1001/jama.271.20.1593

[bib6] Donnelly J, Mack P, Donaldson LA (2001) Follow-up of breast cancer: time for a new approach? Int J Clin Pract 55: 431–433 11594249

[bib7] Elder EE, Kennedy CW, Gluch L, Carmalt HL, Janu NC, Jospeh MG, Donellan MJ, Molland JG, Gillett DJ (2006) Patterns of breast cancer relapse. Eur J Surg Oncol 32: 922–927 16822644 10.1016/j.ejso.2006.06.001

[bib8] Fallowfield L, Cella D, Cuzick J, Francis S, Locker G, Howell A (2004) Quality of life of postmenopausal women in the arimidex, tamoxifen, alone or in combination (ATAC) adjuvant breast cancer trial. J Clin Oncol 22: 4261–4271 15514369 10.1200/JCO.2004.08.029

[bib9] Fowble B, Solin LJ, Schultz DJ, Rubenstein J, Goodman RL (1990) Breast recurrence following conservative surgery and radiation: patterns of failure, prognosis and pathological findings in mastectomy specimens with implications for treatment. Int J Radiat Oncol Biol Phys 19: 833–842 2170305 10.1016/0360-3016(90)90002-2

[bib10] Freedman GM, Anderson PR, Hanlon AL, Eisenberg DF, Nicolaou N (2005) Pattern of local recurrence after conservative surgery and whole-breast irradiation. Int J Radiat Oncol Biol Phys 61: 1328–1336 15817334 10.1016/j.ijrobp.2004.08.026

[bib11] Gulliford T, Opomu M, Wilson E, Hanham I, Epstein R (1997) Popularity of less frequent follow up for breast cancer in randomised study: initial findings from the hotline study. BMJ 314: 174–177 9022429 10.1136/bmj.314.7075.174 PMC2125684

[bib12] Haffty BG, Fischer D, Beinfield M, McKhann C (1991) Prognosis following local recurrence in the conservatively treated breast cancer patient. Int J Radiat Oncol Biol Phys 21: 293–298 2061106 10.1016/0360-3016(91)90774-x

[bib13] Hardman A, Maguire P, Crowther D (1989) The recognition of psychiatric morbidity on a medical oncology ward. J Psychosom Res 33: 235–239 2724199 10.1016/0022-3999(89)90051-2

[bib14] Jack WJL, Kerr GR, Kunkler IH (1998) Long-term follow-up after breast conservation: The Edinburgh experience. The Breast 7: 80–84

[bib15] Khatcheressian JL, Wolff AC, Smith TJ, Grunfeld E, Muss HB, Vogel VG, Halberg F, Somerfield MR, Davidson NE (2006) American Society of Clinical Oncology 2006 update of the breast cancer follow-up and management guidelines in the adjuvant setting. J Clin Oncol 24: 5091–5097 17033037 10.1200/JCO.2006.08.8575

[bib16] Kiebert GM, Welvaart K, Kievit J (1993) Psychological effects of routine follow-up on cancer patients after surgery. Eur J Surg 159: 601–607 8130301

[bib17] Kurtz JM, Spitalier J-M, Almaric R, Brandone H, Ayme Y, Jacquemier J, Hans D, Bressac C (1990) The prognostic significance of late local recurrence after breast conserving therapy. Int J Radiat Oncol Biol Phys 18: 87–93 2298639 10.1016/0360-3016(90)90271-k

[bib18] Mellink W, Holland R, Hendriks J, Peeters P, Rutgers E, Van Daal W (1991) The contribution of routine follow-up mammography to an early detection of asynchronous contralateral breast cancer. Cancer 67: 1844–1848 1848469 10.1002/1097-0142(19910401)67:7<1844::aid-cncr2820670705>3.0.co;2-w

[bib19] Morris S, Corder AP, Taylor I (1992) What are the benefits of routine breast cancer follow up? Postgrad Med J 68: 904–907 1494512 10.1136/pgmj.68.805.904 PMC2399479

[bib20] National Institute for Clinical Excellence. Guidance on Cancer Services: Improving Outcomes in Breast Cancer. Manual Update. http://www.nice.org.uk/page.aspx?o=csgbcguidance (2002). Ref Type: Internet Communication

[bib21] NHS Cancer Screening Programmes (2003). NHS Breast Screening Review (2003) NHS Breast Screening Programme. 2003. England. Ref Type: Pamphlet

[bib22] Paradiso A, Nitti P, Frezza P, Scorpiglione N, on behalf of G.S.Bio.Ca.M (1995) A survey in Puglia: the attitudes and opinions of specialists, general physicians and patients on follow up practice. Ann Oncol 6: s53–s56 8547199 10.1093/annonc/6.suppl_2.s53

[bib23] Passik SD, Dugan W, MvDonald MV, Rosenfeld B, Theobald DE, Edgerton S (1998) Oncologists' recognition of depression in their patients with cancer. J Clin Oncol 16: 1594–1600 9552071 10.1200/JCO.1998.16.4.1594

[bib24] Pennery E, Mallet J (2000) A preliminary study of patients' perceptions of routine follow up after treatment for breast cancer. Eur J Oncol Nurs 4: 138–145 12849643 10.1054/ejon.2000.0092

[bib25] Recht A, Schnitt SJ, Connolly JL, Rose MA, Silver B, Come S, Henderson IC, Slavin S, Harris JR (1989) Prognosis following local or regional recurrence after conservative surgery and radiotherapy for early stage breast carcinoma. Int J Radiat Oncol Biol Phys 16: 3–9 2912955 10.1016/0360-3016(89)90003-5

[bib26] Schunemann H, Willich N (1998) Lymphoedema of the arm after primary treatment of breast cancer. Anticancer Res 18: 2235–2236 9703792

[bib27] The Association of Breast Surgery @ BASO and Royal College of Surgeons of England (2005) Guidelines for the management of symptomatic breast disease. Eur J Surg Oncol 31: S1–S21 10.1016/j.ejso.2005.02.006 15862705

[bib28] The ATAC Trialists' Group (2005) Results of the ATAC (arimidex, tamoxifen, alone or in combination) trial after completion of 5 years' adjuvant treatment for breast cancer. Lancet 365: 60–62 15639680 10.1016/S0140-6736(04)17666-6

[bib29] The GIVIO investigators (1994) Impact of follow-up testing on survival and health-related quality of life in breast cancer patients. A multicenter randomized controlled trial. JAMA 271: 1587–1592 8182811 10.1001/jama.1994.03510440047031

[bib30] The Royal College of Radiologists (1995) The use of imaging in the follow-up of patients with breast cancer. 1995. London. Ref Type: Pamphlet

[bib31] The Steering Committee on Clinical Practice Guidelines for the Care and Treatment of Breast Cancer (1998) Follow-up after treatment for breast cancer. CMAJ 158(suppl 3): S65–S70 9484280

[bib32] Valente SM, Saunders JM, Zichi Cohen M (1994) Evaluating depression among patients with cancer. Cancer Pract 2: 65–71 8055008

